# Caudal block with steroid in the treatment of acute voiding dysfunction and pain caused by sacral herpes zoster: A case report

**DOI:** 10.1097/MD.0000000000020680

**Published:** 2020-06-19

**Authors:** Younghoon Jeon, Yehun Jin

**Affiliations:** a Department of Anesthesiology and Pain Medicine, School of Dentistry, Kyungpook National University, Daegu, Republic of Korea; b Department of Anesthesiology and Pain medicine, Kyungpook National University Hospital, Daegu, Republic of Korea.

**Keywords:** caudal block, herpes zoster, pain, sacral region, steroid, voiding dysfunction

## Abstract

**Rationale:**

Herpes zoster (HZ) involving sacral dermatome is very rare, which can sometimes cause voiding dysfunction.

**Patient concerns:**

A 52-year-old man presented with acute pain and voiding dysfunction, following HZ in his right sacral dermatomes.

**Diagnoses:**

Twenty two days before presentation HZ occurred and 9 days after the onset of the HZ, he had trouble with starting urination and weak urine stream which was managed with tamsulosin 0.4 mg orally once a day and intermittent urinary catheterization. He was treated with 150 mg of pregabalin 2 times a day, tramadol 50 mg 2 times, and acetaminophen 600 mg 2 times a day. However, his pain intensity was 5 on the numerical analogue scale (NRS) from 0 (no pain) to 10 (worst pain imaginable).

**Interventions:**

Fluoroscopy guided caudal block was performed with a mixture of 0.5% lidocaine 10 mL and triamcinolone 40 mg.

**Outcomes:**

One day after the procedure, the pain decreased to 1 on the NRS score. In addition, voiding difficulty greatly improved. Three days after the intervention, the patient reported complete resolution of pain and voiding dysfunction. He currently remains symptom free at a 3-month follow-up.

**Lessons:**

A caudal block with steroid can be an effective option for treatment of acute voiding dysfunction and pain following sacral HZ.

## 1 Introduction

Varicella-zoster virus remains in dormant in the sensory ganglion for decade after a primary chickenpox infection in the childhood. Herpes zoster (HZ) is caused by reactivation of latent varicella-zoster virus, usually resulting in a painful rash in the affected dermatome.^[1]^ However, motor deficits are rare but can occur.^[2,3]^

HZ predominantly involves the thoracolumbar region. Sacral HZ is relatively rare, which can cause acute pain, and voiding dysfunction.^[1,4]^ The possible pathogenic mechanism of this entity includes the spread of HZ virus along the visceral and somatic nerves, leading to cystitis, neuritis, and myelitis.^[4–6]^ The prognosis for bladder dysfunction associated with HZ is usually benign, which successfully returned to within days to weeks. However, some patients with urinary retention need intermittent or indwelling urinary catheterization.^[4–6]^

Interventional procedures such as epidural and paravertebral block with steroid are effective in reducing acute pain from HZ and may prevent postherpetic neuralgia (PHN).^[7,8]^ Here, we reported a patient with acute pain and voiding dysfunction following sacral HZ, which was successfully treated with caudal block using steroid.

## 2 Case report

**Figure 1:**
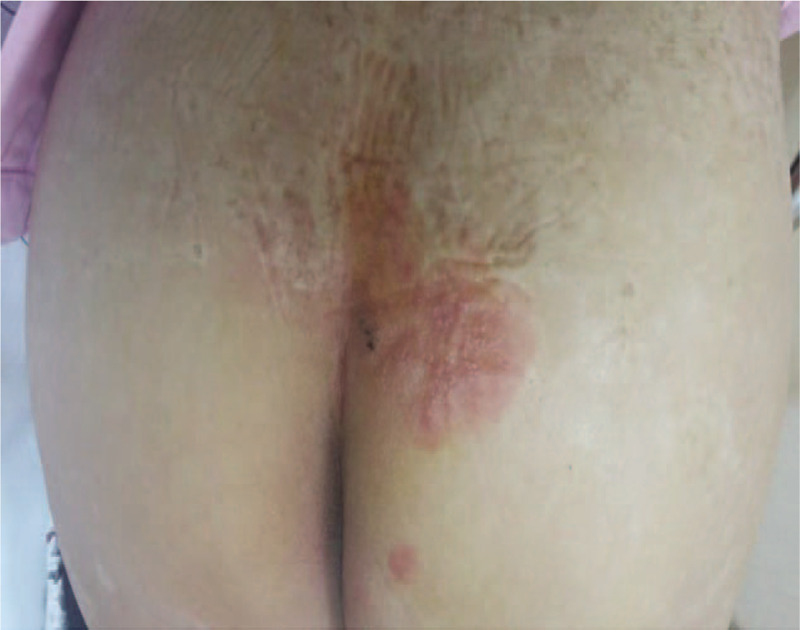
Herpetiform vesicles on an erythematous base involving right S 2–4 in a 52-year-old man.

A 52-year-old man was referred to the pain management center from department of urology with acute pain and voiding dysfunction following HZ in his right sacral dermatomes. Twenty two days before presentation, he noticed a painful herpetic rash on his right buttock and his clinical diagnosis of HZ was made by an urologist. A 7-day course of famciclovir at a dose of 250 mg 3 times a day was initiated. In addition, his initial pain score was 8 rated at an intensity of 8 on the numerical analogue scale (NRS) from 0 (no pain) to 10 (worst pain imaginable). Therefore, he was treated with 150 mg of pregabalin 2 times a day, tramadol 50 mg 2 times, and acetaminophen 600 mg 2 times a day. Nine days after the onset of the rash, he had trouble with starting urination and weak urine stream. He was treated with tamsulosin 0.4 mg orally once a day to aid in voiding. But he intermittently required urethral catheterization with nelaton urinary catheter. His past medical history was unremarkable without history of voiding difficulty. Abdominal x-ray and cystoscopy examination and laboratory tests including urine and blood analysis showed no abnormalities. General physical examination revealed herpetiform vesicles on an erythematous base in his right buttock, penis, and scrotum in the S 2–4 dermatome (Fig. 1). Despite analgesic medications his pain was rated at an intensity of 5 on the NRS. Therefore, fluoroscopy guided caudal block was performed with a mixture of 0.5% lidocaine 10 mL and triamcinolone 40 mg. One day after the procedure, the pain decreased to 1 on the NRS score. In addition, voiding difficulty greatly improved. Three days after the intervention, the patient reported complete resolution of pain and voiding dysfunction and his medications were discontinued. HZ skin lesion was resolved 11 days after caudal block. He currently remains symptom free at a 3-month follow-up. Patient's consent was obtained for the publication of this case.

## 3 Discussion

HZ typically affected sensory ganglion of spinal or cranial nerve, resulting in ipsilateral rash, pain, and sensory change. However, HZ rarely accompanies motor paresis or visceral neuropathies.^[2,3]^

The most common neurological complication of HZ is PHN. HZ usually affects the thoracic dermatomes in up to 60% of all cases.^[1,9]^ Sacral HZ is relatively rare, accounting for 4% to 8% of cases. Approximately 4% of patients with sacral lesion reported urinary voiding dysfunction and constipation.^[4,10]^

Urinary dysfunction in sacral HZ can be caused by cystitis, neuritis, and myelitis.^[4–6]^ In the present case, cystoscopy revealed normal mucosa of bladder with large capacity and there were no laboratory evidences of cystitis, suggesting that voiding dysfunction can be caused by neurologic complication rather than bladder involvement alone. Myelitis in HZ results in spastic bladder along with long tract signs. However, our patient showed no long tract sings. Therefore, it was presumed that sacral HZ caused neuritis, which disrupted parasympathetic motor input to the detrusor muscle, leading to a hypotonic bladder. The patient had trouble with starting urination and weak urine stream, which was treated with terazosin and intermittent urethral catheterization. Tamsulosin, an α_1_-adrenergic antagonist reduces sympathetic tone at the bladder neck, decreasing the detrusor pressure necessary to empty the bladder.^[11,12]^ Antiviral agents such as acyclovir, valacyclovior, and famciclovir are effective to reduce pain and rash of acute HZ and in preventing progression of PHN. In addition, epidural or paravertebral block with steroid can reduce acute HZ pain and prevent PHN.^[3,7,8]^ Steroid in the interventional procedure hastens resolution of neuritis associated with HZ.^[3]^ The prognosis of acute urinary dysfunction following sacral HZ is relatively good. But some patients with sacral HZ often require urinary catheterization. It was reported that resolution of bladder dysfunction took up to 2 months in some cases.^[6]^ In the present case, caudal block with triamcinolone 40 mg alleviated pain and hastened recovery of urinary dysfunction in a patient with sacral HZ. It is hypothesized that epidural administration of steroid is effective to decrease inflammatory response in the affected nerve, leading to rapid recovery of the damaged nerves.

## 4 Conclusion

Sacral HZ is relatively rare. However, it can cause urination difficulty in some patients with sacral HZ, which sometimes requires intermittent or indwelling catheter placement. A caudal block with steroid can be an effective option for treatment of acute voiding dysfunction, and pain following sacral herpes zoster. Physicians should be aware of this entity to provide proper treatments.

## Author contributions

**Data collection and methodology**: Yehun Jin.

**Investigation:** Yehun Jin.

**Supervision:** Younghoon Jeon.

**Writing – original draft & editing**: Younghoon Jeon
